# Practical Medicine

**Published:** 1878-06

**Authors:** 


### PRACTICAL MEDICINE.

METALLOTHERAPY.—Much interest has been aroused by Charcot's investigations into Burq's system of metallotherapy. The latter's theory was that plates of metal placed upon the skin have the property of altering general and special sensation and cutaneous vascular supply. He found that a patient sensitive to one of the metals used—gold, silver, copper, iron, zinc, would not be to another. Burq also claimed that the metal to which a patient was susceptible, was the proper therapeutical agent to be employed in his case.

Charcot got results strikingly corroborative of the above. His patients were marked examples of aggravated hysteria or hystero-epilepsy; and with the metal for which they evinced a peculiar idiosyncrasy, administered *internally*, he succeeded, in four patients experimented upon, in restoring or improving general sensation, increasing muscular power, and bringing back cutaneous circulation. The first case took gold and sodium, the second gold and zinc, the third gold, and the fourth was sensitive to copper, and took the hydrated binoxids, and a mineral water containing a copper salt.

Unfortunately M. Maguan and Dr. Westphal have been unable to obtain like results, and Charcot himself has met with some failures.—*London Medical Record.*

Digiti Mortui. (*N. Y. Medical Recorder*, May 11th, 1878.)—At a recent meeting of the New York Neurological Society, Dr. McBride reported a case of the above disease, with remarks upon its symptoms, course, pathology, and literature. The latter we give for those who may be interested in looking it up. It was first described by Brodie in his lectures on "Local Nervous Disorders," (pub.) 1837. Since then it has been referred to by Huston, 1836; Raymond, 1862, 1872, 1874; Nothnagel, 1866; Estlander, 1870; Fischer, 1875; and by Dr. McLane Hamilton, in the *N. Y. Medical Journal*, Oct. 1874.

The author considered the trouble to be a *vaso-motor* reflex, depending upon increased irritability; that it indicated a nervous system of great mobility, but, otherwise, was without significance. His treatment comprised the direct and induced currents, and alternate hot and cold douches.

Dark and Dark Green Urine from External Use of Carbolic Acid.—M. M. E. Kirmisson, in *La France Médicale*, describes a number of well-marked cases of dark and dark green colored urine, from the external use of carbolic acid. There seems to be no special danger in connection with this appearance. M. K. concludes, however, that as soon as the phenomenon is observed, the use of carbolic acid should be modified. The differential diagnosis between the dark urine from the use of carbolic acid, and the dark urine from certain hepatic troubles, is to be found in the general condition of the patient.

